# Classification, Molecular Characterization, and the Significance of *Pten* Alteration in Leiomyosarcoma

**DOI:** 10.1155/2012/380896

**Published:** 2012-02-15

**Authors:** Allie H. Grossmann, Lester J. Layfield, R. Lor Randall

**Affiliations:** ^1^Department of Pathology, University of Utah, Salt Lake City, UT 84112, USA; ^2^Molecular Medicine Program, University of Utah, Salt Lake City, UT 84112, USA; ^3^Sarcoma Services, Huntsman Cancer Institute, Department of Orthopaedics, University of Utah, Salt Lake City, UT 84112, USA; ^4^Center for Children's Cancer Research, Huntsman Cancer Institute, University of Utah, Salt Lake City, UT 84112, USA

## Abstract

Leiomyosarcoma is a malignant smooth muscle neoplasm with a complicated histopathologic classification scheme and marked differences in clinical behavior depending on the anatomic site of origin. Overlapping morphologic features of benign and borderline malignant smooth muscle neoplasms further complicate the diagnostic process. Likewise, deciphering the complex and heterogeneous patterns of genetic changes which occur in this cancer has been challenging. Preliminary studies suggest that reproducible molecular classification may be possible in the near future and new prognostic markers are emerging. Robust recapitulation of leiomyosarcoma in mice with conditional deletion of *Pten* in smooth muscle and the simultaneous discovery of a novel role for Pten in genomic stability provide a fresh perspective on the mechanism of leiomyosarcomagenesis and promise for therapeutic intervention.

## 1. Introduction

Smooth muscle tumors constitute a spectrum of diseases with wide-ranging clinical behaviors. In general, clinical behavior correlates with patient age, tumor site, histologic appearance, and stage. Leiomyosarcomas (LMSs), the malignant variety, are less common than their benign counterpart, leiomyomas (LMs), and most frequently occur in middle-aged to elderly adults [1]. Children and adolescents generally do not develop LM or LMS, and those rare neoplasms occurring in this population are typically associated with Epstein Barr virus expression, owing to an immunocompromised state [1–3]. Excluding the extremely rare LMS of bone [3], LMS represents approximately 24% of all sarcomas [4] and is, therefore, one of the most common mesenchymal malignancies. The two most frequent sites of origin are the uterus and retroperitoneum, but LMS has been reported in a variety of soft tissue sites, visceral organs, skin, and bone [2, 3]. The diagnostic histopathologic features of smooth muscle tumors are well defined [2]. Architecturally, LM and well-differentiated LMS are composed of bundles and fascicles of cells, intersecting at perpendicular angles. The smooth muscle cells are typically elongate with abundant eosinophilic cytoplasm, cigar-shaped nuclei, and perinuclear vacuoles. Most well-differentiated lesions stain diffusely for actins (smooth muscle actin or muscle-specific actin), and many also stain for desmin, and h-caldesmon. These markers are not specific for smooth muscle, however, and should be interpreted in the context of appropriate clinical and morphologic features. Up to 38% of LMSs will also stain focally for cytokeratins [5], warranting careful discrimination from a sarcomatoid carcinoma and some synovial sarcomas. Epithelioid forms of smooth muscle tumors occur and demonstrate a strong staining pattern for actins, desmin, and h-caldesmon similar to the spindle cell form.

The most widely accepted grading systems for LMS are those defined for all soft tissue sarcomas by the United States National Cancer Institute (NCI) and the French Fédération Nationale des Centres de Lutte Contre le Cancer (FNCLCC) [3]. Both consist of a three-grade scheme in which increasing grade generally correlates with increasing tumor aggressiveness. The NCI system is based on histologic type, cellularity, nuclear atypia, mitotic count, and percent necrosis. Similarly, the FNCLCC system is based on extent of differentiation, mitotic count, and percent necrosis. For many soft tissue sarcomas it is clear that histologic grade strongly predicts outcome [6]. For LMS, however, grade is less predictive and other factors such as neurovascular invasion appear to be prognostic [6–8]. As tumors advance in grade, the obvious morphologic features of smooth muscle and the characteristic immunohistochemical staining can become less prominent, rendering the diagnosis much more challenging. But even well-differentiated smooth muscle tumors can pose significant diagnostic challenges for pathologists. When the full complement of malignant characteristics is lacking, our current understanding of the appropriate classification for predicting behavior is limited. Furthermore, the clinical behavior of smooth muscle tumors is influenced by site of origin, rendering the diagnostic process quite complex. 

## 2. Clinicopathologic Classification of Leiomyosarcoma

For prognostic purposes LMS should be subtyped based on anatomic compartment of origin [1, 2]. Uterine LMS is distinguished from soft tissue LMS, which refers to nonvisceral tumors and includes cutaneous, major vessel, and deep soft tissue [2]. The deep soft tissue tumors can be further divided into retroperitoneal and somatic (peripheral). The cutaneous variety behaves more like a benign tumor when it is strictly limited to the dermis and is probably more aptly termed atypical intradermal smooth muscle neoplasm [9]. Noncutaneous LMS, generally, is an aggressive neoplasm, but noteworthy differences in biologic behavior exist among the subtypes. In a large study of patients with soft tissue LMS [10] the 5- and 10-year survival of subcutaneous LMS (83% and 74%, resp.) was dramatically better than that of deeply-seated tumors (50% and 39%). This same trend, in which the superficial location of subcutaneous LMS predicts increased survival over more deeply seated lesions, was observed in a small study of somatic LMS [11]. The most common form of deep soft tissue LMS arises in the retroperitoneum and portends one of the worst prognoses for LMS. The retroperitoneal tumors typically present as large masses (>10 cm) with involvement of adjacent structures [1]. In more recent retrospective analyses, the median survival time has been reported as 24 months [12] and the overall 5-year survival is less than 50% [13]. LMS of somatic soft tissue has a slightly better prognosis and includes tumors arising in the subcutis, soft tissues of the extremities, and nonretroperitoneal/nonabdominopelvic tissues of the trunk [1, 2, 14]. Given the varied origins of somatic tumors it is reasonable to conclude that there will be additional subtypes described as more studies accumulate. For example, clinicopathologic studies of smooth muscle tumors arising in the soft tissue of the external genitalia [14, 15] and the inguinal region [16] have described unique features which may lead to their distinction. Only very rarely LMS occurs in these locations so it is difficult to ascertain the true behavior of these tumors. It appears for now, though, that genital and inguinal LMSs share some commonalities with other somatic LMSs. For example, somatic LMS often presents at a smaller size than those of the retroperitoneum but remains an aggressive group with a reported 5- year survival rate of 64% [8, 17]. Increasing tumor size, grade, and depth correlates with decreased survival [8, 11, 17–20]. Increasing mitotic index has also been shown to adversely affect prognosis [20, 21], but this correlation has not been true in other studies [8, 11, 17, 18]. Interestingly, at least one-quarter [11] to one-third [17] of somatic LMSs originate from a vessel wall but these lesions are distinguished from the major vessel LMS group, which arises in large vessels, most commonly the inferior vena cava (IVC) [22], have a worse prognosis, and pose uniquely challenging clinical management issues. Even with aggressive surgical resection the five-year survival rate for LMS of the IVC has been reported to be between 33% and 68% [23–29]. A recent retrospective study of 40 LMSs of the IVC documented 5- and 10-year survival rates of 50% and 22% [30]. The authors report that tumor grade does not affect overall prognosis in a multivariate analysis but impaired liver function correlated with the lowest overall survival. In addition, tumors with predominantly intraluminal growth, incomplete resection, and suprahepatic location or right atrial involvement correlate with death within 2 years.

While subtyping LMS by anatomic site is somewhat helpful in predicting outcome, to fully appreciate the spectrum of disease and biologic potential of LMS it is useful to understand that LMS resides on a morphologic and behavioral continuum in which LM, intermediate lesions, and LMS can have overlapping morphologic features. The existence of benign lesions (LM) in deep soft tissue has been controversial. In the last decade attempts have been made at defining these tumors [15, 31], albeit with a healthy measure of caution. The terminology for intermediate smooth muscles tumors (with histopathologic features in between LM and LMS) is complex, reflecting the fact that some of these tumors recur and metastasize, and predicting their behavior is challenging. Because these tumors are rare, there is limited clinical data to correlate behavior with histopathologic features. In addition, the diagnostic criteria for malignancy are site-specific. Since the anatomic compartment helps predict outcome, it is used to help distinguish benign, borderline, and malignant lesions [2, 3, 14, 15, 31]. Borderline lesions may be described with a variety of labels, including, but not limited to, atypical leiomyoma, atypical leiomyoma with potential for recurrence, smooth muscle tumor of *uncertain* malignant potential, and the slightly more ominous, smooth muscle tumor of *low* malignant potential. Criteria for malignancy have been most extensively defined for uterine tumors and consist of a detailed tiered system of weighted histologic criteria including nuclear atypia, presence of coagulative necrosis, and mitotic count [14]. For example, in the setting of no more than mild nuclear atypia and no necrosis, a proliferative index of greater than 9 mitoses per 10 high powered fields (>9/10HPF) renders a borderline diagnostic label and should warrant long-term clinical followup due to the potential for recurrence and even metastasis. True coagulative necrosis lowers the threshold of the minimum mitotic count to <10/10HPF for a borderline tumor and >/= 10/10HPF for LMS. In the absence of necrosis, overt atypia can render a borderline or malignant diagnosis only when mitoses are present. Cytologic atypia and a mitotic index of <10/10HPF yield a borderline diagnosis while a mitotic count of >/= 10/10HPF bumps the diagnosis to LMS. In the absence of mitoses and coagulative necrosis, extremely bizarre nuclear features can occur in the setting of a *symplastic* leiomyoma, now classified as *atypical leiomyoma*. Atypical/symplastic LM is typically characterized by focal, rather than diffuse, cytologic atypia. Importantly, the lack of mitotic activity and necrosis distinguishes it from borderline and malignant lesions. Strict adherence to the criteria for histologic classification is necessary for appropriate diagnosis. Exceptions to these rules exist for nonconventional (epithelioid and myxoid) uterine smooth muscle tumors but are beyond the scope of this paper. Importantly, pathologists must extensively sample smooth muscle tumors to avoid erroneous diagnosis and subsequent inappropriate clinical management. 

Because nuclear atypia, mitotic count, and coagulation necrosis have been useful features for stratifying risk in uterine smooth muscle tumors, these criteria have been applied to smooth muscle tumors from other anatomic locations. As a result, site-specific diagnostic algorithms are emerging [2, 14, 15, 31]. For example, retroperitoneal LM shares many clinical and histopathologic features with uterine LM and while mitotic index is frequently low (<1/50HPF) [32], higher mitotic counts between 3 and 10/50HPF, in the absence of atypia and necrosis, have been reported with long-term followup [31, 32]. These data suggest that similar to uterine LM, mitotic activity alone may not indicate malignancy [2]. Indeed, Hornick and Fletcher [15] have suggested that mitotically active (<10/50HPF) uterine-type (estrogen/progesterone receptor positive by immunohistochemical staining) smooth muscle tumors of the retroperitoneum and abdomen in women are benign when necrosis and significant atypia are absent. Likewise, vulvar and vaginal smooth muscle tumors appear to have a good prognosis in the setting of mild to moderate mitotic activity, and in some cases, even with coexisting nuclear atypia [33–36]. While these lesions may not have metastatic potential, some experts have noted that any mitotic activity or atypia increases the risk of local recurrence up to decades later [15]. Essentially the threshold for definitive malignancy in a smooth muscle tumor of the female external genitalia appears to be high, as seen in the uterus. Hornick and Fletcher [15] define LMS in this region as having at least 3 of 4 malignant features including; (1) mitoses >5/10 HPF, (2) significant atypia, (3) infiltrative margins, or (4) size >5 cm. Accordingly, tumors meeting only 1-2 of the criteria are recommended for the classification of “atypical smooth muscle tumor.” Another potentially hormone-related LM of probable Müllerian origin has recently been described in the inguinal region [16]. Again, low mitotic activity (0-6/10HPF) and mild atypia do not appear to correlate with malignancy in the absence of necrosis. In contrast, mitoses and atypia are significantly less tolerated in smooth muscle tumors of the male external genitalia [14, 15], deep soft tissue of the extremities [2, 14, 15, 31], and retroperitoneal tumors in males [15]. In deep soft tissue, including subcutaneous origin, some studies suggest that *any* level of mitoses is worrisome for potential malignancy [17, 37]. From these various studies it is clear that the only criteria which are uniformly consistent with LM in deep soft tissue are an absolute lack of mitoses, atypia, and necrosis. At present the criteria are not absolute but it appears that any deep soft tissue smooth muscle tumor demonstrating even minimal mitoses or atypia may be reasonably classified as atypical and afforded long-term followup. 

## 3. Promising Discoveries 

The challenge of classifying LMS with histopathologic criteria and predicting outcome, as well as the poor survival associated with this cancer, highlights the need for additional prognostic markers and identification of targets for therapeutic intervention. A variety of approaches have been undertaken which are beginning to provide some insight into the biology of LMS. Recently, microRNA expression profiling has resulted in successful distinction of uterine LMS from LM and normal myometrium (*n* = 10  each) and revealed that LMS has a more primitive, mesenchymal stem cell-like microRNA population than LM [38]. Larger studies will be necessary to determine how reliable and feasible this technique is in distinguishing benign from intermediate and malignant lesions. Within the malignant category, molecular subclasses of LMS are beginning to emerge with the use of RNA expression profiling [39, 40]. With a combined approach of RNA expression profiling, array comparative genomic hybridization (aCGH), and tissue array proteomics, Beck and coauthors [40] identified novel prognostic markers for LMS. The RNA expression array revealed three reproducible clusters of gene expression, classified as groups I–III. Group I showed enrichment of muscle-associated genes, suggesting a more differentiated group of tumors. Expression of these muscle-associated genes, detected in a large tissue array of LMS samples (*n* = 124), predicted prolonged survival. In contrast, expression of the macrophage colony stimulating factor-1 (CSF-1) response pathway, previously identified as a poor prognostic marker in breast cancer [41], predicted poor outcome in the group III subclass of LMS [40, 42]. Although the authors did not report the mean survival times to gauge the actual predicted survival advantage or disadvantage, their results suggest these proteins may be useful as potential prognostic biomarkers in LMS. Interestingly, the aCGH analysis, completed in parallel, correlated closely enough with the RNA expression array that aCGH was able to predict groups I and III. This finding, along with the tissue array data, provides confirmation that at least groups I and III, as determined by RNA expression pattern, are probably meaningful. The ability of the aCGH to align with the expression array data also suggests that within what appears to be uninterpretable chaos in LMS genomes, there may be some hidden clues that hint at the etiology of this cancer.

Unlike the many sarcomas with recurrent chromosomal alterations, LMS demonstrates highly complex, unstable karyotypes (reviewed in [43]). A number of investigators have attempted to find meaning in the complex cytogenetic profiles of LMS and some commonalities have emerged. Specifically, losses of the chromosome 13q and 10q, where the tumor suppressor genes retinoblastoma 1 (*RB1*) and phosphatase and tensin homolog (*Pten),* respectively, reside, are frequently reported alterations in LMS [40, 44–51]. *Pten* point mutations have also been detected in LMS [52–54]. Pten alteration is a common finding in a variety of cancers (reviewed in [55]). The *Pten* gene codes for a lipid and protein phosphatase that antagonizes the lipid kinase activity of phosphatidylinositol 3-kinase (PI3K). Importantly, the lipid phosphatase activity of Pten appears to be nonredundant such that Pten loss consistently results in dysregulation of PI3K, hyperactivation (phosphorylation) of Akt, increased cellular proliferation, and inhibition of apoptosis. PI3K/Akt signaling is facilitated by mammalian target of rapamycin (mTOR), and hyperactivation of this pathway in LMS has been repeatedly reported in human samples [56–58] suggesting that markers such as phosphoAkt may become useful in distinguishing benign from malignant neoplasms. Furthermore, immunohistochemical analysis of the PI3K/Akt pathway activation state could help predict and monitor treatment response and, therefore, guide therapeutic decisions. Preliminary reports on clinical investigations with mTOR inhibitors have shown some activity against LMS. Specifically, one patient with metastatic uterine LMS (out of eight patients with LMS) had a partial response to temsirolimus sustained over 17 months [59]. In another study with temsirolimus, three out of six patients with LMS showed stable disease by RECIST criteria and a partial response by the Choi criteria [60]. Deforolimus has also shown promise. In a larger trial with 221 sarcoma patients, ridaforolimus achieved a clinical benefit rate (defined as a complete or partial response, or stable disease sustained for 16 weeks) of 29% [61]. Of the 57 LMS patients included in this trial, those who received clinical benefit also showed an improvement in overall survival by greater than five months. 

While mTOR inhibitors show potential in the treatment of LMS, the biology of this tumor suggests that mTOR inhibitors will be most effective when used in combination with other agents. *Pten* alterations in LMS, for example, occur in the context of widespread genomic instability. It is likely, therefore, that malignant smooth muscle cells have achieved unregulated growth and metastatic potential through a number of simultaneously functional mechanisms. Molecular studies of human LMS and mouse models of LMS support this idea and may help explain the stepwise progression of changes, involving Pten and other cellular machinery, which lead to malignancy. Cytogenetic studies of LMS have shown that high-grade tumors contain significantly more DNA copy number gains while low-grade tumors contain significantly more copy number losses, suggesting that tumor suppressor loss may be an initiating event and oncogene activation may occur later in malignant progression [49]. DNA copy number gains also increase with increasing tumor size [46] and specific sets of gains are associated with only very large tumors [44]. In keeping with the hypothesis that tumor suppressor loss is an early event that precedes oncogene activation in leiomyosarcoma genesis, Hernando and coauthors [56] reported that conditional deletion of *Pten* in smooth muscle of mice results in hyperactivated Akt signaling, rapid onset of smooth muscle hyperplasia, and LMS. This animal model provides evidence that smooth muscle cells are exquisitely sensitive to the status of Pten function. The authors noted that while the absence of Pten is necessary for tumor development, the smooth muscle hyperplasia that also occurred in these mice, a potential precursor lesion, suggests that Pten deficiency is not sufficient for tumorigenesis. Additional steps required for tumor formation may include *p53* suppression. Indeed, the authors report marked Mdm2 induction in the LMS compared to the nonmalignant smooth muscle of the Pten-null mice. They propose that activated Akt promotes stabilization of Mdm2, thereby inactivating p53. The implication is that Pten loss, with subsequent hyperactivation of PI3K/Akt, could pave the way for genomic instability. This is a provocative concept which is supported by *in vitro* evidence that Akt phosphorylates Mdm2, resulting in ubiquitination and degradation of p53, and that when Pten is present to inhibit Akt, p53 remains functional in mediating sensitivity to DNA damaging agents (reviewed in [62]). Hence, Pten loss may not only help initiate leiomyosarcomagenesis but also contribute to genomic instability and chemoresistance. On the other hand, inhibition of PI3K/Akt signaling, with mTOR or PI3K inhibitors, may have the capacity to sensitize cells to DNA damaging agents, but only in the setting of intact p53 function. If intact p53 function is necessary to achieve the anticancer effects of mTOR and PI3K inhibitors, then careful selection of the appropriate patient population may be warranted.

Shortly before the LMS phenotype was described in the conditionally deleted *Pten* mouse, Shen and coauthors [63] discovered a novel role for Pten in preserving chromosomal integrity through a mechanism independent of its phosphatase activity and ability to regulate of PI3K/Akt signaling. The discovery began with the observation that *Pten*-deficient mouse embryonic fibroblasts spontaneously accumulate a variety of chromosomal aberrations predominated by centromeric breaks. Pten was then found to localize to centromeres through an interaction with centromere binding protein-C (CENP-C). CENP-C is necessary for kinetochore function during mitosis and, therefore, enables appropriate sister-chromatid segregation. Disruption of the interaction of Pten with CENP-C, by either truncation of Pten or point mutation within the C-terminus, leads to chromosome instability. These Pten mutants were derived from known germline mutations in Cowden syndrome patients, and the authors demonstrated that Cowden syndrome lymphoblastoid cells, which are heterozygous for *Pten* mutation, exhibit a high frequency of centromeric breaks. These compelling data suggest that this form of mutant *Pten*, similar to *TP53* gain of function mutants [64], can function as an oncogene, rather than a tumor suppressor. In tumors, point mutations in the N-terminal phosphatase domain of *Pten* are well documented and mutations in the C-terminal domain account for approximately 40% of the reported mutations [65].

Pten dysfunction is potentially one of many mechanisms contributing to the development of genomic instability in LMS. Genomic instability is a major molecular feature of LMS progression, and other genes have been implicated. For example, *TP53* mutation has been documented in 24–39% of LMS [66, 67], is associated with higher-grade tumors [66], and appears to be restricted to LMS because they do not occur in LM [67]. In a recent study, Beck and coauthors [40] showed frequent loss of chromosome 16q24 in a particular molecular subset of LMS. This region harbors the Fanconi anemia complementation group A (*FANCA*) gene, which codes for one member of a large multiprotein complex that coordinates with breast cancer, early onset (Brca) proteins in the DNA damage signaling and repair process [68]. Xing and coauthors [69] demonstrated that 29% (25 out of 85) uterine LMSs show immunohistochemical loss of Brca1. In the same study, conditional deletion of *Brca1* in the developing Müllerian duct of the mouse did not lead to tumor formation but *p53* deletion resulted in the development of malignant smooth muscle tumors of the uterus. Concomitant deletion of *Brca1* and *p53* resulted in a more accelerated phenotype. Conditional deletion of *p53* and *Brca1* can also induce LMS in the ovary [70]. *Mdm2* amplification, an alternative means of downregulating p53, has been reported in 14% of soft tissue LMS [71, 72]. These data indicate a variety of mechanisms leading to DNA repair defects in LMS. Importantly, new therapeutic agents which exploit DNA repair defects in cancer cells have been developed.

The parallel discoveries of chromosome 16q24, *FANCA* [40], and *Brca1* [69] loss in LMS suggest defective double strand break (DSB) repair and, therefore, potential vulnerability to poly (ADP-ribose) polymerase (PARP) inhibitors (reviewed in [73]). PARP1 and PARP2 proteins are essential for the resolution of single-strand DNA breaks by the base excision repair machinery. When PARP inhibitors are employed in the context of defective DSB repair by homologous recombination (HR) repair mechanisms, such as with Brca mutation, cells become extremely sensitive to DNA damaging agents. PARP inhibitors create synthetic lethality in this situation by shutting down a second mechanism of DNA repair. Interestingly, Pten has been linked to DSB repair by regulating the expression of RAD51, a HR repair protein, and Pten loss is associated with defective DSB repair [63, 74]. Furthermore, PARP inhibitors generate synthetic lethality in Pten-deficient tumors [74–76]. These data suggest that in subsets of LMS with DSB repair defects, which may include Pten deficiency, PARP inhibitors may be able to sensitize these tumors to DNA damaging agents.

## 4. Conclusions

The era of personalized cancer medicine has begun, and recent advancements in our understanding of LMS indicate that tailored therapy for this disease is in sight. Molecular and proteomic analysis is beginning to uncover biomarkers which may improve our ability to classify smooth muscle tumors and predict clinical outcome. Furthermore, the parallel and profound discoveries that Pten is a critical guardian of genomic integrity and initiator of LMS in mice provide a mechanism of tumorigenesis and potential Achilles heel. The high frequency of *Pten* alteration in cancer, together with its role in genomic stability and synthetic lethal relationship with PARP inhibition, suggests that PARP inhibitors may find widespread use as anticancer agents [73], including in LMS. Screening for alterations in *Pten* and other genes which regulate or directly participate in DSB repair may help guide the use of PARP inhibitors in LMS and other cancers. In fact, as new classes of DNA repair inhibitors emerge, the need may eventually arise to evaluate the entire cellular DNA damage response machinery in every tumor subject to systemic treatment. Likewise, the effectiveness of mTOR and PI3K inhibitors may be dependent on *TP53* status. A complete evaluation of *Pten* and *TP53* is notoriously labor intensive due to the wide variety of mechanisms by which these genes can be altered. One of the challenges in the future of personalized cancer care will be to develop appropriate, reliable, and affordable laboratory-based clinical tests with reasonable turn-around times. We are optimistic this is an achievable goal with next-generation sequencing approaches to molecular profiling. Emerging molecular technologies may also help reveal new insights into the biology of LMS and improve the effectiveness of therapeutic intervention.
